# Analysis of ALPS‐Index: Difference in Type 2 Diabetes Mellitus With or Without Mild Cognitive Impairment and Its Relationship With Hippocampal Microstructure

**DOI:** 10.1002/brb3.70672

**Published:** 2025-07-21

**Authors:** Ziyu Diao, Xuan Huang, Die Shen, Kun Wang, Jiahe Wang, Kui Zhao, Chen Zhao, Zidong Cao, Xin Tan, Shijun Qiu

**Affiliations:** ^1^ The First Clinical Medical College Guangzhou University of Chinese Medicine Guangzhou China; ^2^ MR Research Collaboration Siemens Healthineers Guangzhou China; ^3^ Department of Radiology The First Affiliated Hospital of Guangzhou University of Chinese Medicine Guangzhou China; ^4^ State Key Laboratory of Traditional Chinese Medicine Syndrome Guangzhou China

**Keywords:** ALPS‐index, cognition, glymphatic system, hippocampal microstructure, magnetic resonance imaging (MRI), type 2 diabetes mellitus

## Abstract

**Introduction:**

Type 2 diabetes mellitus (T2DM) with cognitive impairment has a high incidence rate globally, and there is a need to investigate the relationship among the glymphatic system, hippocampus microstructure, and cognition in T2DM. The present study aims to delineate changes in the perivascular space index (ALPS‐index) among T2DM patients with different cognitive states and investigate any possible correlation between the ALPS‐index and the diffusive indicators of the bilateral hippocampi in T2DM. In addition, we seek to identify specific cognitive domains with substantial correlation with the ALPS‐index in the general population.

**Methods:**

A total of 113 participants were recruited, comprising 37 T2DM patients with normal cognitive function (DMNC), 39 T2DM patients with mild cognitive impairment (DMMCI), and 37 healthy controls (HC). Clinical information, neuropsychological assessments, and experienced multimodal magnetic resonance imaging scans were recorded from all the participants. A noninvasive method was applied to obtain all ALPS‐index measures, such as the left, right, and average ALPS‐index, along with diffusive indicators of the bilateral hippocampi, comprising fractional anisotropy (FA), mean diffusivity (MD), axial diffusivity (AD), and radial diffusivity (RD).

**Results:**

A statistically marginal difference in the average ALPS‐index was noted among HC, DMNC, and DMMCI groups. According to binary logistic regression analysis results, the average ALPS‐index significantly altered the cognitive function in T2DM. Partial correlation analyses revealed a positive association between the average ALPS‐index and FA, as well as a negative association with MD and RD in the bilateral hippocampi of the T2DM groups. In the general population, partial correlation analysis indicated that the average ALPS‐index correlated with the auditory verbal learning test (AVLT) immediate recall scores.

**Conclusion:**

In summary, our findings demonstrated that glymphatic system function in the brain progressively deteriorates with worsening cognitive impairment among T2DM patients. Moreover, T2DM significantly disrupts the relationship patterns between the glymphatic system and bilateral hippocampal microstructure. Thus, the mean ALPS‐index may act as a novel neuroimaging biomarker for assessing cognitive function in T2DM.

## Graphical abstract

This study demonstrated that glymphatic system function in the brain progressively deteriorates with worsening cognitive impairment among T2DM patients. Moreover, T2DM significantly disrupts the relationship patterns between the glymphatic system and bilateral hippocampal microstructure.

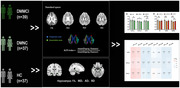

## 1 Introduction

Type 2 diabetes mellitus (T2DM) is a chronic metabolic disorder characterized by enduring hyperglycemia and insulin resistance (Li et al. 2021). The 10th edition of the Diabetes Atlas, released by the International Diabetes Federation (IDF), projects that by 2045, the global population affected by diabetes will be approximately 700 million, among which the majority (90%–95%) will be those diagnosed with T2DM (Ehtewish et al. 2022). Cognitive impairment is a common comorbidity of diabetes, with meta‐analyses indicating that approximately 45% of individuals with diabetes are affected by mild cognitive impairment (MCI) (Liu et al. 2020; You et al. 2021; Militaru et al. 2024). MCI is a precursory phase to Alzheimer's disease (Petersen et al. 2018). As such, identifying a common underlying mechanism causing MCI among T2DM patients can significantly help in formulating specific interventions and treatment plans, geared toward reducing cognitive deterioration.

One promising avenue of research points toward the glymphatic system, which is responsible for waste removal and regulation of solute balance through the cerebrospinal fluid (CSF) flow within the brain. To some degree, this system acts as the innate self‐cleansing process of the brain, promoting health. It majorly involves continuous exchange between CSF and interstitial fluid (ISF). CSF moves from the subarachnoid space into the perivascular spaces around the arteries before diffusing into the brain tissue via aquaporin‐4 (AQP4) water channels located at the terminal extensions of astrocyte cells (Iliff and Nedergaard 2013). As the CSF becomes saturated with metabolic byproducts and ISF within the brain, it is ultimately directed into the perivascular spaces encircling the veins, subsequently merging with the peripheral lymphatic system (Iliff et al. 2012). AQP4, an important component of the brain glymphatic system, is essential for improving CSF flow and elimination of waste products within the cerebral parenchyma (Benveniste et al. 2019). AQP4 expression is significantly reduced in T2DM, causing impaired glymphatic system performance (Zhang et al. 2016). Therefore, disruptions in the glymphatic system may promote cognitive decline among individuals with T2DM.

Magnetic resonance imaging (MRI) is widely used for assessing the human brain's glymphatic system (Taoka and Naganawa 2020). Previous studies used the intrathecal injection or intravenous (IV) administration of gadolinium‐based contrast agents (GBCA) as a tracer to investigate the glymphatic system (Ringstad et al. 2017; Naganawa et al. 2019). However, this method is invasive and unsuitable for individuals allergic to the contrast agents. In 2017, a noninvasive novel technique was introduced in the field of MRI, that is, diffusion tensor imaging analysis along the perivascular space (DTI‐ALPS) techniques, which have leveraged diffusion MRI (dMRI) to assess the glymphatic system activity via the ALPS‐index. The ALPS‐index acts as a metric that evaluates the diffusion ability alongside the periventricular regions encircling deep venous structures compared to the diffusion across the primary fiber tracts (Taoka et al. 2017). Due to its high reproducibility and stability, the ALPS‐index has been widely applied in recent research on degenerative neurological disorders, such as Alzheimer's disease (Hsu et al. 2022) and Parkinson's disease (Chen et al. 2021; Shen et al. 2022), ischemic stroke (Toh and Siow 2021), multiple sclerosis (Carotenuto et al. 2022), obstructive sleep apnea–hypopnea syndrome (Xiong et al. 2024) and T2DM (Yang et al. 2020). Recent investigations into the glymphatic system within the context of T2DM have been scarce, primarily focusing on its relationship with insulin resistance, the disease's chronicity, and cognitive function (Yang et al. 2020; Tuerxun et al. 2024; Yu et al. 2024). One clear limitation of the above studies was that they did not perform group‐level studies based on cognitive status in T2DM (Yu et al. 2024). However, we believe that investigating the levels of the glymphatic system across various cognitive states in T2DM would help in elucidating the etiology of cognitive deficits associated with T2DM.

Notably, cognition is related to the structure and function of the hippocampus in T2DM patients (den Heijer et al. 2003; Van Bussel et al. 2016; Li et al. 2023). DTI is a good technique that indicates the microstructure integrity in the hippocampus by measuring the diffusion of water molecules in the tissues, whose indicators primarily include fractional anisotropy (FA), mean diffusivity (MD), axial diffusivity (AD), and radial diffusivity (RD) (Zhou et al. 2011; Bao et al. 2018). Additionally, one rat study suggested that T2DM could obstruct the drainage of ISF, specifically affecting regions including the hippocampus, which might be a factor in the development of cognitive deficits (Jiang et al. 2016). With the progression of diabetes, there is an increase in the deposition of fibrin and platelets in the blood vessels of the hippocampal region, along with perivascular AQP4 loss and beta‐amyloid deposition. This indicates that the microstructure of the hippocampus may be associated with the role of the glymphatic system (Boyd et al. 2024). Thus, there is a need to investigate the relationship between the glymphatic system in the brain and the microstructure of the hippocampus among patients with T2DM.

On the basis of these findings, this investigation proposes the following hypotheses: (1) The ALPS‐index shows a progressive decline corresponding to the severity of cognitive impairment in patients with T2DM. (2) The ALPS‐index shows significant associations with the microstructural integrity of the hippocampus, as measured by diffusion metrics.

## 2 Methods

### 2.1 Participants

In this cross‐sectional study, a total of 76 patients diagnosed with T2DM (39 with DMMCI and 37 with DMNC) were recruited from The First Affiliated Hospital of Guangzhou University of Chinese Medicine between October 2022 and April 2024. Moreover, a healthy control (HC) group comprising 37 community‐dwelling participants was included, rigorously matching the patient group by gender, age, and education level. All participants met the inclusion criteria of being right‐handed, aged 30–70 years, and having at least 6 years of formal education. Our sample size of 113 participants adhered to the required sample size of 102 calculated by G*Power (version 3.1.9.7).

The diagnostic process of T2DM was guided by the standards proposed by the American Diabetes Association (ElSayed et al. 2023). The inclusion criteria for MCI included (1) memory decline complaints; (2) 16 < Montreal Cognitive Assessment (MoCA) score  <  26 (Nasreddine et al. 2005; Trzepacz et al. 2015; Carson et al. 2017); (3) preserved general functional abilities. In the T2DM group, individuals who met the criteria for MCI were categorized into the DMMCI group, whereas the others were categorized into the DMNC group. Participants in the HC group were considered to have normal cognitive function (MoCA ≥ 26). The exclusion criteria included the following: (1) patients with other neurological or psychiatric disorders that could affect cognitive function including dementia, major depressive disorder, and so forth; (2) those with intracranial organic diseases, such as cerebral infarction, cerebral hemorrhage, and cerebral aneurysm, and so forth; (3) patients with other endocrine disorders, including hyperthyroidism, hyperparathyroidism, polycystic ovary syndrome, Cushing's syndrome and so on; (4) contraindications for MRI scanning.

### 2.2 Clinical and Neuropsychological Measurements

The body mass index (BMI) and arterial blood pressure of all enrolled participants were meticulously documented, along with the disease duration, specifically for patients with T2DM. A thorough battery of laboratory investigations was then conducted on all participants. This involved a comprehensive assessment of fasting plasma glucose (FPG), fasting insulin (FINS), glycosylated hemoglobin (HbA1c), homeostasis model assessment of insulin resistance (HOMA‐IR) low‐density lipoprotein (LDL), high‐density lipoprotein (HDL), total cholesterol (TC), and triglycerides (TG).

All participants underwent an extensive suite of neuropsychological evaluations, including the MoCA, auditory verbal learning test (AVLT), grooved pegboard test (GPT), digit span test (DST), clock‐drawing test (CDT), digit symbol substitution test (DSST), and Trail‐Making Test Part A (TMT‐A). These cognitive assessment tools collectively assess global cognition, memory, attention, motor skills, visuospatial abilities, processing speed, and executive function. Each participant thoroughly undertook a prearranged sequence of comprehensive assessments.

All scale assessments were conducted by two trained radiologists. All evaluators underwent standardized prior training guided by neuropsychologists to ensure the accuracy of the assessments. In addition, a double‐blind evaluation process was implemented to reduce possible subjective biases from the evaluators.

### 2.3 MRI Acquisition

To improve the accuracy of data, the MAGNETOM Prisma 3T MRI scanner (Siemens, Germany) furnished with a 64‐channel head coil was used to obtain a comprehensive dataset of brain dMRI through the diffusion spectrum imaging (DSI) technique. The imaging technique employed was an echo planar imaging with multiple slices along the posterior–anterior axis, using 99 diffusion gradient directions and 12 *b* values, including 0, 300, 350, 650, 950, 1000, 1350, 1650, 1700, 2000, 2700, and 3000 s/mm^2^. Additionally, the parameters included the following: TR/TE = 4200/72 ms, FOV = 220 mm × 220 mm, slice thickness = 2 mm, voxel size = 2 × 2 × 2 mm^3^, NEX = 1, and acquisition time = 7 min and 31 s. Another b0 image with an opposite phase encoding direction was acquired to adjust for EPI distortions. The parameters for the 3D T1‐weighted imaging (T1WI) sequence included the following: inversion time TI = 1100 ms, TR = 2530 ms, TE = 2.98 ms, FOV = 256 mm × 256 mm, slice thickness = 1 mm, voxel size = 1 × 1 × 1 mm^3^, flip angle = 7°, with a total of 192 sagittal slices.

### 2.4 ALPS‐Index

**FIGURE 1:**
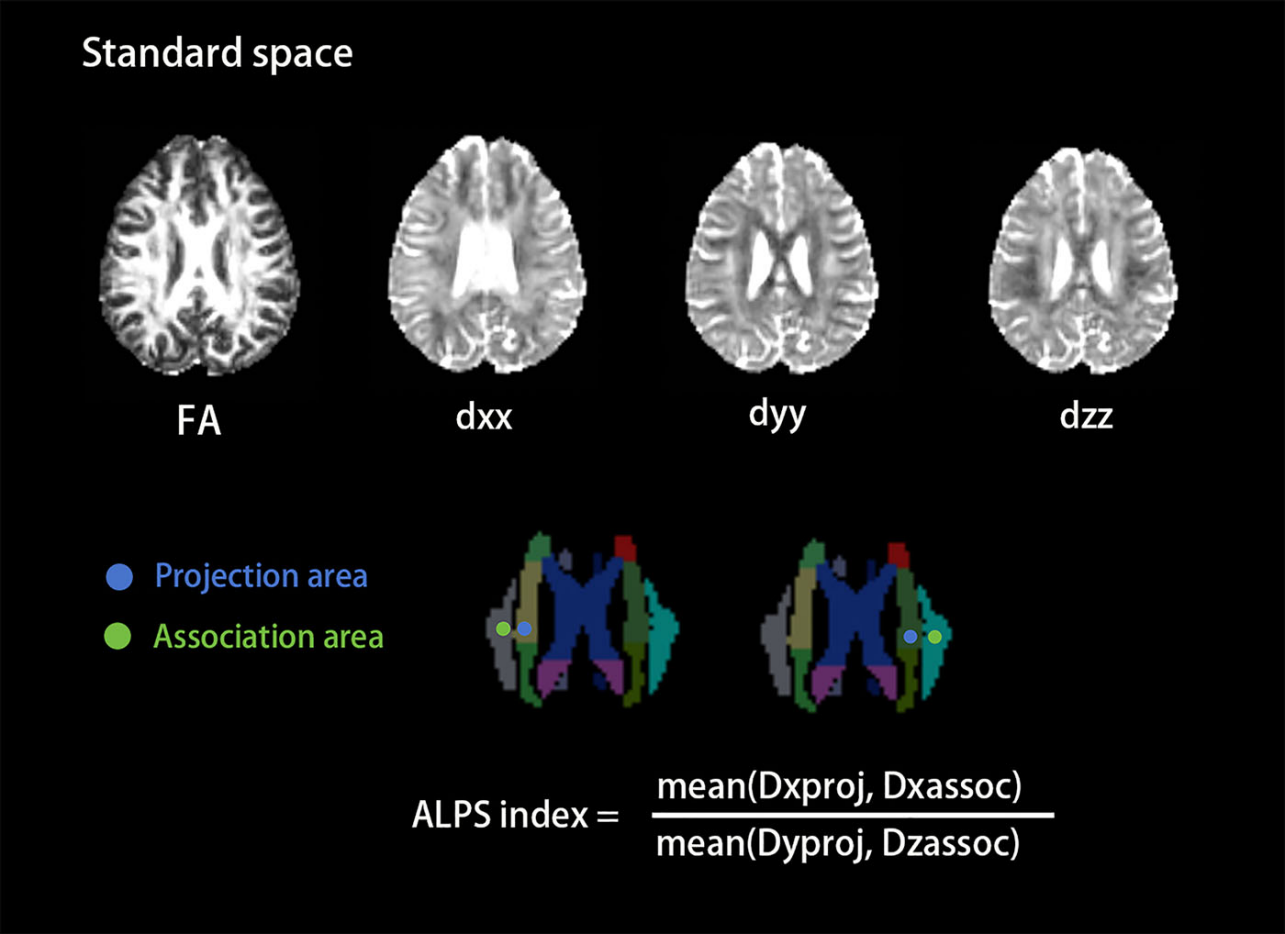
Calculation of the ALPS‐index. FA map, *dxx* map, *dyy* map, and *dzz* map in MNI152 standard space after spatially normalized. *dxx*: diffusivity maps in the direction of the *x*‐axis; *dyy*: diffusivity maps in the direction of the *y*‐axis; *dzz*: diffusivity maps in the direction of the *z*‐axis. The placement of the ROIs (blue and green circles with a diameter of 5 mm) was determined on JHU atlas. FA, fractional anisotropy.

*MATLAB (Version, R2022a)* and *FSL (Version, 6.0.1)* software were used for the meticulous processing of MRI data to obtain precise brain structural and functional information. Below are the comprehensive steps for data processing:

1. Data preprocessing: The *b* values below 1000 were extracted from the original dMRI dataset and DICOM‐NIFTI format transformation to ensure the accuracy of subsequent analyses.
2. Distortion correction: The “Eddy” and “Topup” commands were employed to address possible distortions arising from magnetic susceptibility variations, eddy current effects, and subject head movement.
3. Image enhancement: Skull stripping was performed, and a layer of CSF surrounding the brain was removed to enhance image quality.
4. Diffusion coefficient map generation: We obtained diffusion coefficient maps along the *x*‐axis (right–left; *dxx*), *y*‐axis (anterior–posterior; *dyy*), and *z*‐axis (inferior–superior; *dzz*), as well as FA maps (Figure 1).
5. Spatial normalization: The FA map in the subject was aligned to the JHU‐ICBM‐FA‐1 mm in the Montreal Neurological Institute (MNI) 152 template, and the transformation matrix was applied to normalize the *x*, *y*, and *z*‐axis diffusion coefficient maps.
6. Region‐of‐interest (ROI) localization: In MNI space, referring to the white matter regions delineated by the JHU atlas, the coordinates for the ROIs included the following: The left projection fiber was located at (116,109,99), the left association fiber at (126,109,99), the right projection fiber at (64,109,101), and the right association fiber at (54,109,101), with all ROIs having a diameter set to 5 mm (Figure 1).
7. Diffusivity analysis: Water diffusivity along the perivenous space was computed, including the *x*‐axis diffusivity within the projection fiber (*Dx*projc) and association fiber (*Dx*assoc), as well as diffusivity in other non‐fiber running directions, including the *y*‐axis diffusivity within the projection fiber (*Dy*proj) and the *z*‐axis diffusivity within the association fiber (*Dz*assoc).
8. ALPS‐index calculation: bilateral ALPS‐index and the average of bilateral ALPS‐index. The calculation formula was: ALPS‐index = mean (*Dx*proj, *Dx*assoc)/mean (*Dy*proj, *Dz*assoc) (Figure 1).

### 2.5 Bilateral Hippocampal Diffusive Indicators

1. DICOM‐NIFTI format transformation.
2. Distortion correction: Similar process to ALPS‐index.
3. Image enhancement: Similar process to ALPS‐index.
4. Diffusion coefficient map generation: We obtained FA, MD, AD, and RD maps.
5. Spatial normalization: Nonlinear registration to the MNI standard space was performed using T1WI. The warp was applied to align FA, MD, AD, and RD maps to the MNI standard space.
6. Anatomical automatic labeling 90 (AAL90) atlas application: The AAL90 atlas was used to acquire FA, MD, AD, and RD values of the bilateral hippocampi.

### 2.6 Statistical Analysis

Statistical analyses were primarily carried out using *R (version, 4.4.1)* and *SPSS (version, 26.0)* software. Shapiro–Wilk test and Levene's test were performed to assess the normal distribution of data and variance homogeneity. Data conforming to both criteria were subjected to a one‐way analysis of variance (ANOVA), followed by Tukey's post hoc analysis. For data violating these assumptions, the Kruskal–Wallis test was applied, with Dunn's test for subsequent post hoc analysis. The Benjamini–Hochberg method for false discovery rate (FDR) was utilized to adjust the post hoc *p* values. Gender comparison was performed using the Chi‐square test. Disease duration was analyzed using the Kruskal–Wallis test for independent non‐parametric groups.

A general linear model (GLM) was employed to ascertain differences in the ALPS‐index between DMNC and DMMCI groups, adjusting for duration, FPG, and LDL as covariates. Binary logistic regression was performed to investigate the relationship between the ALPS‐index and T2DM patients, with significance defined as *p* values below 0.05 (two‐tailed).

For bilateral hippocampal diffusive indicators, the two‐sample *t*‐test was used when the data adhered to the assumptions of normal distribution and homogeneity of variances; the Mann–Whitney *U* test was used in the absence of these assumptions. In both the T2DM and HC groups, partial correlations were conducted to elucidate the relationships between the ALPS‐index and the FA, MD, AD, and RD indicators of the bilateral hippocampi. In the general population, partial correlations were used to assess the correlation between the ALPS‐index and cognitive performance scores in different domains with gender, age, education, BMI, FPG, HbA1c, LDL, and HDL acting as covariates with statistical significance set at *p* < 0.05.

## 3 Results

### 3.1 Demographics, Clinical Characteristics, and Neuropsychological Measurements

This study enrolled 113 participants. Table 1 summarizes demographic, clinical, and cognitive data of all participants. We found no significant difference in age, sex, or years of education among the three groups (all *p* > 0.05). Unlike HC and DMNC, patients with DMMCI performed significantly worse in most of neurological tests, including MoCA, AVLT, GPT, DSST, and TMT‐A (*p* < 0.05).

**TABLE 1:** Demographic and clinical data and cognitive performance of all participants.

	HC(*n* = 37)	DM(*n* = 76)		Statistics	*p* value
		DMNC(*n* = 37)	DMMCI(*n* = 39)		
Gender (male/female)	11/26	15/22	19/26	*X* ^2^ = 2.868	0.238
Age (year)	51(45,59)	49(45,55)	53(48.5,59)	*H* = 2.32	0.314
Education (year)	10(8,15)	12(9,15)	9(8,12)	*H* = 4.36	0.113
Duration (year)	NA	4(2,12)	4(2,8.5)	*H* = 161.02	**<0.001**
BMI (kg/mm^2^)	22.94(21.3,24.12)	23.73(22.04,25.62)	23.39(20.79,26.01)	*H* = 1.62	0.444
SBP (mmHg)	124.22 ± 12.96	120.38 ± 14.08	127.26 ± 15.75	*F* = 2.19	0.117
DBP (mmHg)	82.59 ± 8.95	81.81 ± 9.13	82.1 ± 8.74	*F* = 0.07	0.93
HbA1c (%)	5.7(5.5,5.9)	7.7(6.5,9.1)	7.4(6.4,9.15)	*H* = 52.15	**<0.001** ^a^, ^b^
FPG (mmol/L)	5.14(4.8,5.51)	6.73(3.74,10.79)	6.6(6,7.84)	*H* = 55.28	**<0.001** ^a^, ^b^, ^c^
FINS (μIU/mL)	7.65(5.74,10.4)	7.7(6.5,9.1)	6.94(4.745,12.03)	*H* = 1.79	0.409
HOMA‐IR	1.72(1.41,2.36)	2.28(1.39,3.78)	2.21(1.45,4.41)	*H* = 2.42	0.299
LDL (mmol/L)	3.05(2.72,3.31)	3.39(2.92,3.79)	2.9(2.285,3.4)	*H* = 6.96	**0.031** ^c^
HDL (mmol/L)	1.47(1.2,1.84)	1.12(0.99,1.43)	1.14(0.94,1.45)	*H* = 10.48	**<0.01** ^a^, ^b^
TG (mmol/L)	1.28(0.89,1.58)	1.41(1.03,2.35)	1.45(1.095,2.14)	*H* = 2.56	0.278
TC (mmol/L)	4.92(4.6,5.58)	5.12(4.55,5.41)	4.74(3.745,5.42)	*H* = 3.08	0.214
MoCA	28(27,28)	27(27,28)	23(21,25)	*H* = 80.78	**<0.001** ^b^, ^c^
AVLT‐immediate	22.78 ± 4.45	21.68 ± 4.36	18.97 ± 4.11	*F* = 7.9	**<0.001** ^b^, ^c^
AVLT‐5min	8.62 ± 1.53	8.32 ± 1.8	6.56 ± 2.07	*F* = 14.29	**<0.001** ^b^, ^c^
AVLT‐delay	9(8,10)	8(7,9)	6(5,7.5)	*H* = 22.31	**<0.001** ^a^, ^b^, ^c^
AVLT‐recall	12(11,12)	12(11,12)	11(10,12)	*H* = 14.11	**0.001** ^b^, ^c^
GPT‐R (s)	62(55,69)	72(64,88)	83(69,98.5)	*H* = 28.34	**<0.001** ^a^, ^b^
GPT‐L (s)	67(60,75)	77(69,89)	87(78,113)	*H* = 33.84	**<0.001** ^a^, ^b^, ^c^
DST‐forward	8(7,9)	8(6,9)	8(6,8)	*H* = 1.29	0.525
DST‐backward	4(3,5)	5(4,5)	4(3,5)	*H* = 5.45	0.066
CDT	4(3,4)	4(4,4)	4(3,4)	*H* = 4.7	0.095
DSST	45(36,54)	39(30,50)	32(26.5,41.5)	*H* = 15.81	**<0.001** ^a^, ^b^, ^c^
TMT‐A (s)	42(31,54)	43(37,47)	48(41.5,68)	*H* = 9.15	**0.01** ^b^, ^c^

*Note*: Data are expressed as the mean ± standard deviation (SD) and median (interquartile range (IQR)). *p* value for comparison among HC, DMNC, and DMMCI by ANOVA or Kruskal–Wallis *H* tests only listed *p*<0.05 for multiple comparisons. Values shown in bold are those that reached statistical significance (*p *< 0.05).

Abbreviations: AVLT, auditory verbal learning test; BMI, body mass index; CDT, clock‐drawing test; DBP, diastolic blood pressure; DMMCI, T2DM patients with mild cognitive impairment; DMNC, T2DM patients with normal cognitive function; DSST, digit symbol substitution test; DST, digit span test; FBG, fasting blood glucose; FINS, fasting insulin; FPG, fasting plasma glucose; GPT, grooved pegboard test; HbA1c, hemoglobin A1c; HC, healthy control; HDL, high‐density lipoprotein; HOMA‐IR, homeostatic model assessment of insulin resistance; LDL, low‐density lipoprotein; MoCA, Montreal cognitive assessment; SBP, systolic blood pressure; TC, total cholesterol; TG, triglyceride; TMT‐A, Trail‐Making Test Part A.

^a^ Post hoc analysis showed a significant group difference between HC and DMNC.

^b^ Post hoc analysis showed a significant group difference between HC and DMMCI.

^c^ Post hoc analysis showed a significant group difference between DMNC and DMMCI.

### 3.2 DTI‐ALPS‐Index

#### 3.2.1 The Difference Among HC, DMNC, and DMMCI Groups

**FIGURE 2:**
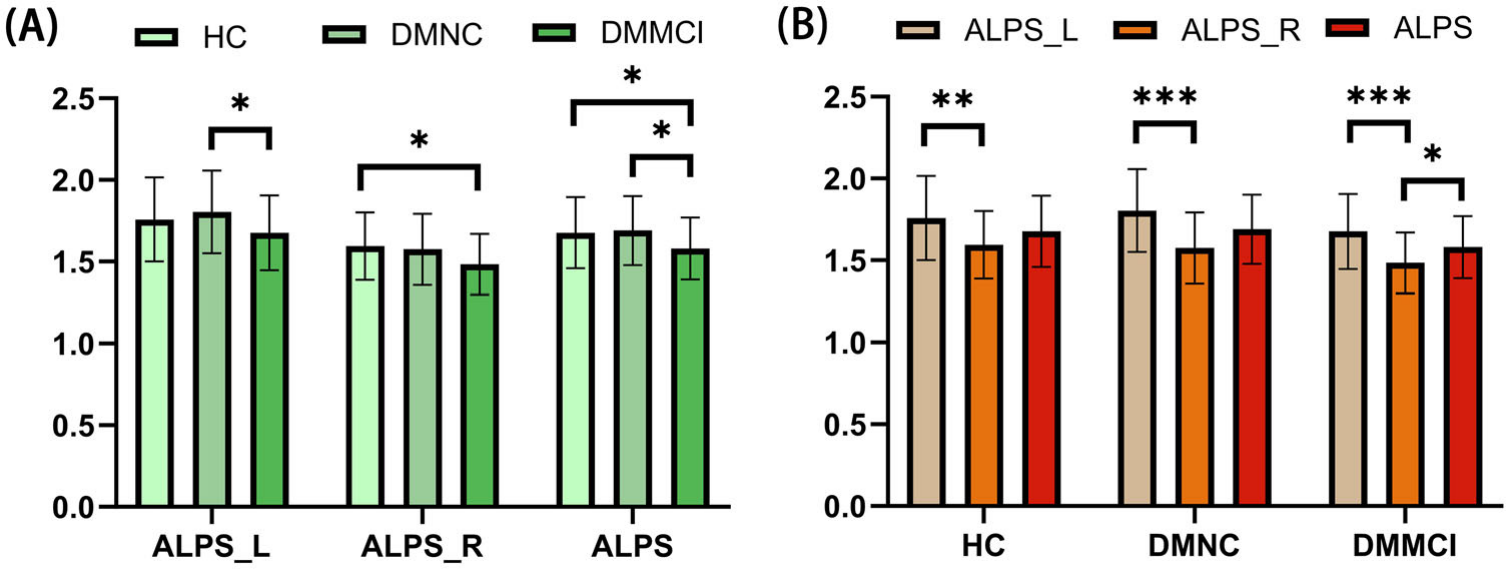
Comparison of all ALPS‐indices among the three groups and differences of three groups across all ALPS‐indices. A.Comparison of all ALPS‐indices among the three groups. B.Differences of three groups across all ALPS‐indices. *p < 0.05; **p < 0.01; ***p < 0.001.

The HC, DMNC, and DMMCI groups showed a near‐significant trend in the left ALPS‐index (*p* = 0.077). Post hoc analysis revealed that the left ALPS‐index of the DMMCI group was significantly lower than that of the DMNC group (*p* = 0.026) (Figure 2A). The HC, DMNC, and DMMCI groups also exhibited significant differences in the right ALPS‐index (*p* = 0.043). Post hoc analysis revealed that the right ALPS‐index of the DMMCI group was significantly lower than that of the HC group (*p* = 0.019) (Table 2, Figure 2A). Furthermore, the three groups showed a marginal effect in the average ALPS‐index (*p* = 0.056). Post hoc analysis with *p* value correction revealed that the average ALPS‐index of the DMMCI group was significantly lower than that of the HC (*p* = 0.04) and the DMNC (*p* = 0.044) groups (Figure 2A). However, no significant difference was found between HC and DMNC groups in all of ALPS‐index. We also found no significant difference observed in the post hoc analysis with *p* value correction for both the left ALPS‐index and the right ALPS‐index.

**TABLE 2:** Comparison of all ALPS‐indices among the three groups.

		DM(*n* = 76)				DM	
	HC(*n* = 37)	DMNC(*n* = 37)	DMMCI(*n* = 39)	Statistics	*p* value	Statistics	*p* value
ALPS_L	1.76 ± 0.26	1.80 ± 0.25	1.68 ± 0.23	*F* = 2.62	0.077^b^	*F* = 6.784	**0.011**
ALPS_R	1.59 ± 0.21	1.58 ± 0.22	1.48 ± 0.19	*F* = 3.24	**0.043** ^a^	*F* = 2.613	0.11
ALPS	1.72(1.51,1.81)	1.68(1.54,1.83)	1.58(1.48,1.74)	*H* = 5.75	0.056^a^, ^b^	*F* = 5.702	**0.02**

*Note*: ALPS_L: the left ALPS‐index; ALPS_R: the right ALPS‐index; the average ALPS‐index: ALPS. The meaning of the bolded values represents reaching the significant threshold (*p* < 0.05) with statistical significance. The statistics and *p* value showed the intergroup differences in three groups, as determined using the ANOVA or KW analysis. The statistics and *p* value below DM represent the intergroup differences between the DMNC and DMMCI, as determined using the GLM analysis.

Abbreviations: DMMCI, T2DM patients with mild cognitive impairment; DMNC, T2DM patients with normal cognitive function; HC, healthy control.

^a^ Post hoc analysis showed a significant group difference between HC and DMNC.

^b^ Post hoc analysis showed a significant group difference between DMNC and DMMCI.

A GLM analysis was conducted due to the statistical differences in disease duration, FPG, and LDL between the DMNC and DMMCI groups, which may influence the results. The GLM showed that the DMMCI group and DMNC group had differences in the left ALPS‐index and average ALPS‐index with the duration, FPG, and LDL as covariates (ALPS_L: *p* = 0.011; ALPS_R: *p* = 0.110; ALPS: *p* = 0.020) (Table 2).

**TABLE 3:** Comparison of the three groups among all ALPS‐indices.

	ALPS_L	ALPS_R	ALPS	Statistics	*p* value
HC	1.76 ± 0.26	1.59 ± 0.21	1.68 ± 0.22	*F* = 4.72	**0.011** ^a^
DMNC	1.80 ± 0.25	1.58 ± 0.22	1.69 ± 0.21	*F* = 9.3	**<0.001** ^a^
DMMCI	1.71(1.55,1.83)	1.48(1.33,1.67)	1.58(1.47,1.75)	*H* = 16.23	**<0.001** ^a^, ^b^

Abbreviations: DMMCI, T2DM patients with mild cognitive impairment; DMNC, T2DM patients with normal cognitive function; HC, healthy control. The meaning of the bolded values represents reaching the significant threshold (*p* < 0.05) with statistical significance.

^a^ Post hoc analysis showed a significant group difference between ALPS_L and ALPS_R.

^b^ Post hoc analysis showed a significant group difference between ALPS_R and ALPS.

#### 3.2.2 The Difference Among Left, Right, and Average ALPS‐Index

Three ALPS‐index had statistical differences in HC, DMNC, and DMMCI groups. Post hoc analysis with *p* value correction left ALPS‐index was higher than the right ALPS‐index in all of the groups (HC: *p* = 0.008, DMNC: *p* < 0.001, DMMCI: *p* < 0.001). Besides, the average ALPS‐index was higher than the right ALPS‐index in DMMCI (*p* = 0.047) (Figure 2B, Table 3).

#### 3.2.3 Binary Logistic Regression Analyses

**FIGURE 3:**
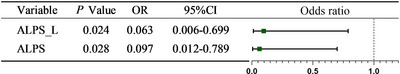
Binary logistic regression analyses for the association between ALPS‐index and cognition in T2DM group. ALPS_L: the left ALPS‐index; the average ALPS‐index: ALPS. OR, odds ratio.

To improve clinical interpretation, binary logistic regression analyses were performed and the percentage form of the all of ALPS‐index was utilized in the logistic regression analysis. The results of logistic regression revealed that the left ALPS‐index was a strong protective factor for the T2DM group, and the reduction of the left ALPS‐index (%) was associated with the risk of MCI in the T2DM group (OR 0.063, 95% CI: 0.006–0.699, *p* = 0.024). The average ALPS‐index was also a strong protective factor for T2DM, and the reduction of the average ALPS‐index (%) was linked to the risk of MCI in the T2DM group (OR 0.097, 95% CI 0.012–0.782, *p* = 0.028) (Figure 3).

### 3.3 Bilateral Hippocampal Diffusion Indices

We observed no difference in the bilateral hippocampal diffusion indices between the T2DM group and the HC group (Table S1).

### 3.4 Partial Correlation Analysis

#### 3.4.1 Between All ALPS‐Indexes and Diffusive Indicators of Bilateral Hippocampi in T2DM

**FIGURE 4:**
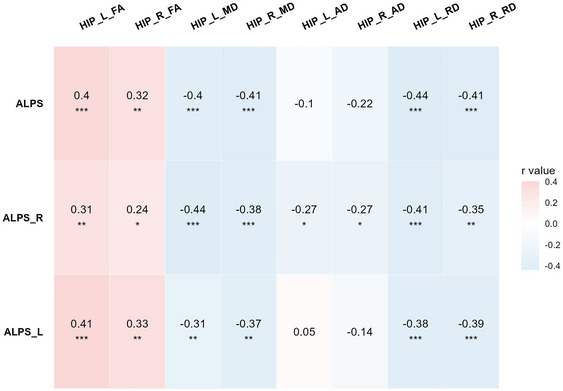
The partial correction analysis between all ALPS‐indices and diffusive indicators of bilateral hippocampi in T2DM group. Partial correlation with gender, age, education and BMI as covariates. *p < 0.05; **p < 0.01; ***p < 0.001.

Partial correlation analysis in the T2DM group showed that the left ALPS‐index, right ALPS‐index, and average ALPS‐index correlated with the diffusive indicator of bilateral hippocampi with gender, age, education, and BMI as covariates (Figure 4). Partial correlation analysis positively correlated with the average ALPS‐index and FA in the left (L: *r* = 0.40, *p* < 0.001) and right (R: *r* = 0.32, *p* < 0.01) hippocampi, as well as negative correlations with MD (L: *r* = −0.40, *p* < 0.001; R: *r* = −0.41, *p* < 0.001) and RD (L: *r* = −0.44, *p* < 0.001; R: *r* = −0.41, *p* < 0.001) in the bilateral hippocampi of the T2DM group. No correlation was found between all of the ALPS‐index and the diffusive indicator of bilateral hippocampi in the HC group with gender, age, education, and BMI as covariates (Table S1).

#### 3.4.2 Between All of the ALPS‐Index and Neuropsychological Indicators in All Participants

**FIGURE 5:**
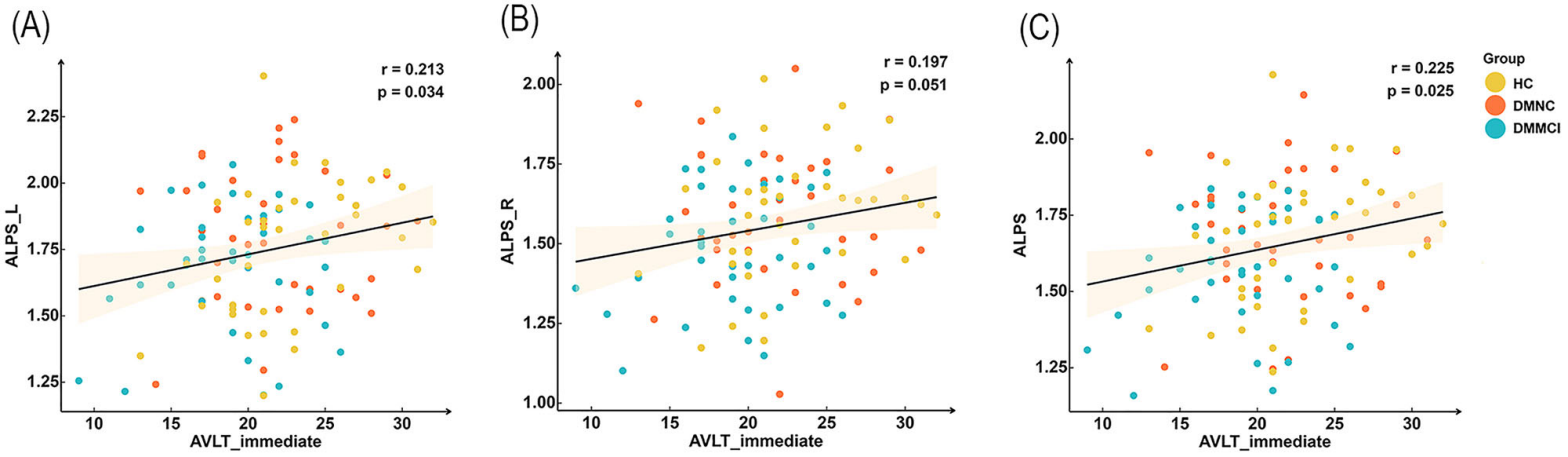
The partial correction analysis between all ALPS‐indices and AVLT‐immediate scores in all participants. Partial correlation with gender, age, education, SBP, LBP, FPG, FINS, HbA1c, HOMA‐IR, BMI, LDL, HDL, TG, and TC served as covariates. DMMCI, T2DM patients with mild cognitive impairment; DMNC, T2DM patients with normal cognitive function; HC, healthy control. A. The partial correction analysis between the left ALPS‐index and AVLT‐immediate score. B. The partial correction analysis between the right ALPS‐index and AVLT‐immediate score. C. The partial correction analysis between the average ALPS‐index and AVLT‐immediate score.

Partial correlation analysis in all subjects showed a positive correlation between the left ALPS‐index and AVLT immediate score (*r* = 0.213, *p* = 0.034, Figure 5A), between the right ALPS‐index and AVLT immediate score (*r* = 0.197, *p* = 0.051, Figure 5B) and between the average ALPS‐index and AVLT immediate score (*r* = 0.225, *p* = 0.025, Figure 5C) with gender, age, education, SBP, LBP, FPG, FINS, HbA1c, HOMA‐IR, BMI, LDL, HDL, TG, and TC acting as covariates. Nonetheless, no partial correlation was observed between all of ALPS‐index and AVLT‐5min, AVLT‐20min, AVLT‐recall, GPT‐R, GPT‐L, TMT‐A, CDT, DSST, DST‐forward, DST‐backward, and MoCA (Table S2).

## 4 Discussion

The present study utilized one‐way ANOVA to evaluate variations in the ALPS‐index among individuals with DMNC, DMMCI, and HC, geared toward exploring the differences among the three groups. Our findings revealed that the average ALPS‐index may act as a reliable and stable metric for assessing the glymphatic system. We also found a downward trend in the average ALPS‐index for DM patients, and the glymphatic system in DMMCI patients may be more compromised than that in DMNC and HC individuals. Partial correlation analysis has also linked the ALPS‐index with hippocampal performance in T2DM. Similarly, we found a positive correlation between the ALPS‐index and the AVLT immediate score in the general population, further emphasizing the relevance of the ALPS‐index to cognitive function.

Despite a downward trend, no significant statistical difference in the average ALPS‐index was observed between DMNC patients and HC. This corroborates the findings of previous studies that reported significant differences, indicating a compromised glymphatic system function in T2DM (Tuerxun et al. 2024). After adjusting for disease duration, FPG, and LDL levels, the average ALPS‐index in the DMMCI group remained significantly decreased, suggesting a more impaired glymphatic system function. Insulin resistance in T2DM reduces the brain's sensitivity to insulin, impairing the glymphatic system and causing amyloid‐beta (Aβ) accumulation within the neuronal insulin signaling pathway (Talbot 2014). Lower ALPS‐index values have been associated with accelerated Aβ deposition, increased risk of amyloid‐positive transition, clinical progression, and fast cognitive decline (Huang et al. 2024), indicating that the high average ALPS‐index is a protective factor for cognition in T2DM. A positive correlation observed between AVLT‐immediate scores and the ALPS‐index in the general population implies that the efficiency of the glymphatic system in clearing ISF may be important in preserving immediate memory performance in the general population. Our binary logistic regression analysis revealed that the average ALPS‐index is a strong protective factor for cognition in T2DM. Similar results are found in the cognition in vascular cognitive impairment (VCI) and aging‐related cognitive decline (Song et al. 2022; Wang et al. 2023). Therefore, the average ALPS‐index is a strong protective factor for cognition in T2DM and associated with immediate memory performance in the general population.

This work used both left and right ALPS‐index measurements, as well as their average to evaluate the three groups. Consequently, the right ALPS‐index tends to be lower than the left, in line with previous studies that documented brain lateralization effects on the glymphatic system's function (Morita et al. 2023; Zhao et al. 2023). Noteworthy, lateralization may be associated with the brain's structural asymmetry (Duboc et al. 2015). Most current studies rely on the average ALPS‐index to evaluate the glymphatic system (Andica et al. 2023; Huang et al. 2024). One study comparing various ALPS indices (Taoka et al. 2022), along with our findings, corroborated the stability of this approach, hence suggesting that the average ALPS‐index is a useful indicator.

The hippocampus is a region with both white and gray matter (Shahid et al. 2022); dMRI analysis can be used to investigate its microstructure (Cappellani et al. 2014; Mak et al. 2017; Jiang et al. 2020). A breakdown of microstructure in gray matter would be observed as lower FA and higher MD (Jiang et al. 2020). Lower cognitive performance causes microstructure breakdown in T2DM (Xiong et al. 2016; Groeneveld et al. 2018). Changes in FA and MD occur before volumetric changes, hence making them sensitive indicators of microstructural alterations (Hong et al. 2010). The lack of differences in diffusive indicators between the DM and HC groups may be attributed to the calculation encompassing the entire hippocampal region, which could mask diverse tissue compositions within various hippocampal subregions (Kalus et al. 2004). Therefore, future research should investigate the relationship between the ALPS‐index and diffusive indicators of hippocampal subregions in T2DM.

Although the changes across the diffusive indicators of bilateral entire hippocampal regions are too subtle to verify the statistical significance between groups, we found that the lower ALPS‐index is associated with lower FA, higher MD, and higher RD to the bilateral hippocampi in T2DM. One animal study revealed that T2DM inhibits the ability of the glymphatic system to remove ISF in the hippocampus, potentially contributing to cognitive deficits in T2DM rats (Jiang et al. 2016). A systematic review revealed that T2DM causes compromised blood–brain barrier (BBB) integrity, elevated apolipoprotein E (APOE) expression, and intensified cognitive deterioration due to metabolic dysregulation from the impaired glymphatic system function (Kim et al. 2018). Although we did not achieve the ideal results similar to those from animal experiments, we can conclude that T2DM alters the relationship between ALPS and the hippocampus. Thus, there is a need for a large sample size to carry out an in‐depth analysis of the relationship between the ALPS index and hippocampal microstructure.

## 5 Limitations

First, the participant pool of our study was modest, and the study was confined to a single‐site, cross‐sectional investigation. Second, the potential influence of T2DM on the ALPS‐index through alterations in sleep patterns remains undetermined due to the absence of sleep status assessment in the current investigation. Considering that T2DM is a condition characterized by polyuria, the resultant nocturnal polyuria could potentially impair sleep quality. Third, the present study did not include the collection of treatment protocols. Besides, all subjects with T2DM were from a single hospital, receiving medications, and insulin treatment to varying extents.

## 6 Conclusion

In conclusion, we discovered a slightly low average ALPS‐index in patients with DMNC and a lower average ALPS‐index in those with DMMCI, suggesting that glymphatic system function will gradually be impaired with cognitive impairment in T2DM. The high average ALPS‐index is a strong protective factor for cognition in T2DM. Furthermore, the average ALPS‐index is linked to the microstructure of bilateral hippocampi in the T2DM group. Our findings ascertain that the average ALPS‐index can represent the entire glymphatic system in T2DM patients to some extent, potentially acting as an emerging neuroimaging biomarker for cognitive decline in T2DM.

## Author Contributions

**Ziyu Diao**: investigation, conceptualization, data curation, methodology, formal analysis, writing – original draft, writing – review and editing, visualization. **Xuan Huang**: investigation, data curation, formal analysis, writing – review and editing, validation. **Die Shen**: investigation, data curation, formal analysis, writing – original draft. **Kun Wang**: data curation, investigation, formal analysis, writing – original draft. **Jiahe Wang**: data curation, investigation, formal analysis, writing – original draft. **Kui Zhao**: methodology, software. **Chen Zhao**: methodology, software. **Zidong Cao**: software, methodology. **Xin Tan**: data curation. **Shijun Qiu**: project administration, funding acquisition, resources, supervision, writing – original draft, writing – review and editing.

## Ethics Statement

Ethical approval for this study was granted by the Medical Research Ethics Committee of the First Affiliated Hospital of Guangzhou University of Chinese Medicine (NO. K‐2023‐146).

## Consent

All participants provided written informed consent prior to participation in the study.

## Conflicts of Interest

The authors declare no conflicts of interest.

## Publisher's Note

All claims expressed in this article are solely those of the authors and do not necessarily represent those of their affiliated organizations, or those of the publisher, the editors, and the reviewers. Any product that may be evaluated in this article, or claim that may be made by its manufacturer, is not guaranteed or endorsed by the publisher.

## Peer Review

The peer review history for this article is available at https://publons.com/publon/10.1002/brb3.70672

## Supporting information




**Supplementary Tables**: brb370672‐sup‐0001‐SuppMat.docx

## Data Availability

The raw data supporting the conclusions of this article will be made available by the authors, without undue reservation.
