# Supplementary material for: Analysis of ALPS‐Index: Difference in Type 2 Diabetes Mellitus With or Without Mild Cognitive Impairment and Its Relationship With Hippocampal Microstructure
Source: Brain Behav. 2025 Jul 21;15(7):e70672. doi: 10.1002/brb3.70672 (PMC12277526; doi:10.1002/brb3.70672)
Supplement: Supplementary file 1 — Supplementary Tables: brb370672‐sup‐0001‐SuppMat.docx [file BRB3-15-e70672-s001.docx]

Supplementary Material

**Supplementary Table 1. The difference in bilateral hippocampus diffusive indicators between HC and DM groups.**

|  | HC(n=37) | DM(n=76) | Statistics | *P* Value |
| --- | --- | --- | --- | --- |
| HIP_L_FA | 0.34(0.33,0.35) | 0.35(0.32,0.36) | W=1509 | 0.53 |
| HIP_R_FA | 0.35±0.03 | 0.35±0.03 | t=0.09 | 0.93 |
| HIP_L_MD(×10^3^) | 0.63(0.62,0.64) | 0.63(0.61,0.64) | W=1357 | 0.77 |
| HIP_R_MD(×10^3^) | 0.63(0.61,0.63) | 0.62(0.61,0.64) | W=1437.5 | 0.85 |
| HIP_L_AD(×10^3^) | 0.87±0.02 | 0.87±0.02 | t=0.09 | 0.92 |
| HIP_R_AD(×10^3^) | 0.87±0.02 | 0.87±0.02 | t=0.93 | 0.35 |
| HIP_L_RD(×10^3^) | 0.51(0.5,0.52) | 0.51(0.49,0.52) | W=1356 | 0.76 |
| HIP_R_RD(×10^3^) | 0.5(0.49,0.52) | 0.5(0.48,0.52) | W=1393.5 | 0.94 |

HIP: hippocampus; L: left; R: right; FA: fractional anisotropy; MD: mean diffusivity; AD: axial diffusivity; RD: radial diffusivity

**Supplementary Table 2. The correlations between all of ALPS-index and neuropsychological indicators in all participants**

|  | ALPS_L | | ALPS_R | | ALPS | |
| --- | --- | --- | --- | --- | --- | --- |
|  | *r* | *P* | *r* | *P* | *r* | *P* |
| MoCA | 0.118 | 0.246 | 0.158 | 0.118 | 0.148 | 0.143 |
| AVLT-5min | 0.01 | 0.921 | 0.061 | 0.548 | 0.036 | 0.726 |
| AVLT-20min | 0.11 | 0.279 | 0.105 | 0.302 | 0.118 | 0.246 |
| AVLT-recall | -0.046 | 0.65 | -0.015 | 0.881 | -0.035 | 0.727 |
| GPT-R | -0.073 | 0.475 | -0.132 | 0.192 | -0.108 | 0.286 |
| GPT-L | -0.085 | 0.405 | -0.097 | 0.338 | -0.099 | 0.331 |
| TMT-A | 0.021 | 0.839 | 0.056 | 0.579 | 0.04 | 0.695 |
| CDT | 0.15 | 0.139 | -0.05 | 0.626 | 0.067 | 0.508 |
| DSST | 0.1 | 0.325 | 0.103 | 0.312 | 0.111 | 0.275 |
| DST-forward | 0.03 | 0.771 | -0.001 | 0.99 | 0.017 | 0.864 |
| DST-backward | 0.146 | 0.149 | 0.142 | 0.161 | 0.158 | 0.119 |

ALPS_L: the left ALPS-index; ALPS_R: the right ALPS-index; the average ALPS-index: ALPS

; MoCA: montreal cognitive assessment; AVLT: auditory verbal learning test; GPT: grooved pegboard test; DST: digit span test; CDT: clock-drawing Test; DSST: digit symbol substitution test; TMT-A: trail making test-A
